# Simultaneous three-dimensional myocardial T1 and T2 mapping in one breath hold with 3D-QALAS

**DOI:** 10.1186/s12968-014-0102-0

**Published:** 2014-12-20

**Authors:** Sofia Kvernby, Marcel Jan Bertus Warntjes, Henrik Haraldsson, Carl-Johan Carlhäll, Jan Engvall, Tino Ebbers

**Affiliations:** Division of Cardiovascular Medicine, Department of Medical and Health Sciences, Linköping University, Linköping, Sweden; Center for Medical Image Science and Visualization (CMIV), Linköping University, Linköping, Sweden; SyntheticMR AB, Linköping, Sweden; Department of Clinical Physiology, County Council of Östergötland, Linköping, Sweden; Division of Media and Information Technology, Department of Science and Technology, Linköping University, Linköping, Sweden

**Keywords:** Relaxation time, T1 mapping, T2 mapping, Three-dimensional, Myocardium, Cardiovascular magnetic resonance

## Abstract

**Background:**

Quantification of the longitudinal- and transverse relaxation time in the myocardium has shown to provide important information in cardiac diagnostics. Methods for cardiac relaxation time mapping generally demand a long breath hold to measure either T1 or T2 in a single 2D slice. In this paper we present and evaluate a novel method for 3D interleaved T1 and T2 mapping of the whole left ventricular myocardium within a single breath hold of 15 heartbeats.

**Methods:**

The 3D-QALAS (3D-quantification using an interleaved Look-Locker acquisition sequence with T2 preparation pulse) is based on a 3D spoiled Turbo Field Echo sequence using inversion recovery with interleaved T2 preparation. Quantification of both T1 and T2 in a volume of 13 slices with a resolution of 2.0x2.0x6.0 mm is obtained from five measurements by using simulations of the longitudinal magnetizations Mz. This acquisition scheme is repeated three times to sample k-space. The method was evaluated both in-vitro (validated against Inversion Recovery and Multi Echo) and in-vivo (validated against MOLLI and Dual Echo).

**Results:**

In-vitro, a strong relation was found between 3D-QALAS and Inversion Recovery (R = 0.998; N = 10; p < 0.01) and between 3D-QALAS and Multi Echo (R = 0.996; N = 10; p < 0.01). The 3D-QALAS method showed no dependence on e.g. heart rate in the interval of 40–120 bpm. In healthy myocardium, the mean T1 value was 1083 ± 43 ms (mean ± SD) for 3D-QALAS and 1089 ± 54 ms for MOLLI, while the mean T2 value was 50.4 ± 3.6 ms 3D-QALAS and 50.3 ± 3.5 ms for Dual Echo. No significant difference in in-vivo relaxation times was found between 3D-QALAS and MOLLI (N = 10; p = 0.65) respectively 3D-QALAS and Dual Echo (N = 10; p = 0.925) for the ten healthy volunteers.

**Conclusions:**

The 3D-QALAS method has demonstrated good accuracy and intra-scan variability both in-vitro and in-vivo. It allows rapid acquisition and provides quantitative information of both T1 and T2 relaxation times in the same scan with full coverage of the left ventricle, enabling clinical application in a broader spectrum of cardiac disorders.

## Background

In daily clinical practice cardiovascular magnetic resonance (CMR) settings are generally chosen to highlight intensity contrast between tissues. By varying parameters such as the echo time, repetition time and flip angle it is possible to create different contrast weighted images. The absolute intensity in contrast weighted images is defined on an arbitrary scale that differs from one examination to another. Image interpretation thus relies on comparisons with surrounding tissue. Recently it has become feasible to obtain a quantitative measurement of the signal intensity corresponding to the absolute value of the longitudinal relaxation (T1), transverse relaxation (T2) and proton density in many organs [1]. This enables numerical comparison between different tissue compositions and hence the objective detection of pathological processes by measuring the deviation from the range of normal values. Another important advantage of these quantitative images is the application of synthetic CMR, which uses the absolute parameters T1 and T2 to synthesize any T1-weighted or T2-weighted contrast image [2]. In this way conventional images can be interpreted with simultaneous access to the quantitative parameters from the same acquisition.

For cardiac applications, both quantitative T1 maps and quantitative T2 maps have been shown to provide important information. T1 maps have been successfully used to identify the area of myocardial infarction and to assess acute and chronic myocardial infarction [3]. Diffuse tissue changes such as diffuse fibrosis may be quantified non-invasively using T1 mapping where remote, normal, myocardium is not accessible for comparison [4-6]. Quantitative T2-mapping techniques have been validated in myocardial ischemia and edema [7,8] with robust results and increased accuracy in comparison with the commonly used T2-weighted images. In the assessment of edema in ischemic and non-ischemic heart disease, even native T1 mapping has provided helpful information [9]. Native T1- and T2-mapping techniques have also been validated for the determination of the area at risk after acute myocardial infarction [10]. Mapping of both relaxation parameters has potential for improving the differentiation between myocardium affected by various diseases and normal myocardium.

Various methods are available to quantify the absolute parameters T1 and T2 in the heart. Due to cardiac motion and time constraints of the cardiac cycle, several difficulties are associated with these methods. The major drawback with the existing quantitative methods is the long imaging time. An inversion recovery sequence can be used to estimate values of T1 in the heart. To allow full magnetization recovery between the radio frequency inversion pulses a relaxation period of four to five times T1 is necessary. Since T1 often has a value exceeding the duration of the cardiac cycle, the clinical applicability of the method is limited. Look and Locker described a multi point approach [11], where the relaxation curve is sampled multiple times after the initial inversion pulse, which contributes to accelerate the acquisition. The drawback with the original Look Locker (LL) -method is that it uses continuous data acquisition and not selective data acquisition in a specific time frame of the cardiac cycle. Messroghli et al. presented, a now established, modified LL inversion recovery sequence (MOLLI) [12] which uses selective data acquisition and multiple LL experiments. The MOLLI sequence acquires in its original format one single slice of the heart in 17 heartbeats, which is a limitation to its clinical use since multiple breath holds may be required to cover the myocardium. A shortened version of MOLLI has also been presented with good agreement to the original version [13], but this is still a 2D mapping method providing a single slice of the heart in one breath hold.

Three-dimensional T1-mapping methods of the heart do exist but generally suffer from long imaging time. Recently Coniglio et al. presented a 3D Inversion Recovery T1 mapping method of the heart using both ECG and respiratory triggers with accurate T1 estimates [14]. This free-breathing method was reported to take 5–8 minutes for six slices. Warntjes et al. presented a 3D single breath hold method for T1, but this approach is only appropriate at short T1 times, i.e. after administration of Gd contrast agent [15].

A multi echo sequence can be used to estimate values of T2 in the heart. Such a sequence often requires several echo time points and thus several repetitions to get a good signal to noise ratio in the T2 map and to sample enough data, which then becomes time consuming. T2 mapping methods using T2-preparation pulses exists and they appear to be robust and fast but they still only provide a single 2D slice map [16].

In this paper we present and evaluate a novel method for 3D interleaved T1 and T2 quantification of the whole left ventricular myocardium within a single breath hold of 15 heartbeats.

## Method

### Pulse sequence design

**Figure 1:**
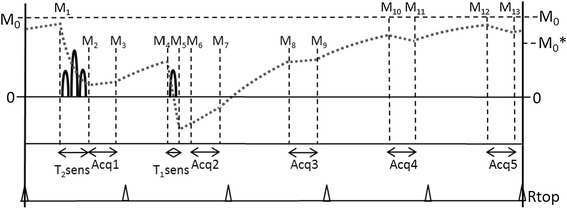
**Schematic overview of the proposed acquisition kernel.** Five cardiac-triggered acquisitions (Acq1- Acq5) are performed during end-diastole. Prior to the first acquisition a T_2_-sensitizing phase decreases the M_z_ magnetization proportional to the T_2_ relaxation. Prior to the second acquisition a T_1_-sensitizing phase is applied to invert the M_z_ magnetization. No sensitizing phases are applied before the other acquisitions. The typical M_z_ magnetization evolution is displayed as the grey dotted line. At each interval the magnetization is labeled as M_1_-M_13_.

The sequence is based on a cardiac triggered 3D spoiled fast gradient echo sequence, Turbo Field Echo, and named 3D-QALAS (3D-QuAntification using an interleaved Look-Locker Acquisition Sequence with T2 preparation pulse). The sequence block, described in Figure 1, can be divided into two phases, one T2 sensitizing phase and one T1 sensitizing phase, with five segmented acquisitions that are run three times during a single breath hold.

The T2 sensitizing phase starts with a non slice-selective 90-degree RF pulse, followed by two 180-degree adiabatic RF refocusing pulses in a time of 50 ms. During this time the magnetization in the xy plane decreases with the T2 relaxation time. After the last 180-degree pulse, a 90-degree RF pulse is applied to tilt the magnetization vector back to the Mz axis.The result of the T2 sensitizing phase is that T2 relaxation is encoded on the Mz axis. After the T2 sensitizing phase a 3D turbo field echo data acquisition is performed.

The T1 sensitizing phase consists of a non slice-selective 180-degree 3D adiabatic inversion pulse followed by gradient spoiling. After an inversion time of 100 ms, a second acquisition is performed. During the following T1 relaxation, three more acquisitions are performed in consecutive heartbeats.

The acquisition is hence performed once after the T2-prep pulse and four times after the T1sensitizing inversion pulse (5 heartbeats). The acquisitions use a flip angle of 5°, a TFE-factor of 90, an echo time of 1.2 ms and a repetition time of 2.6 ms. This acquisition scheme is repeated three times to sample k-space, resulting in a breath hold of 15 heartbeats.

The number of required k-space points is reduced due to the use of elliptical k-space filling. The center of k-space is sampled first and the periphery last (low-high order). The acquisition is accelerated using a sensitivity encoding (SENSE) reduction factor of 2 in the phase encoding (y) direction and a factor of 1.2 in the slice (z) direction. To minimize artifacts from cardiac motion the acquisition window is restricted to 230 ms in end-diastole by using cardiac ECG triggering. The resolution of the 3D acquisition is 2.0 mm, 2.0 mm and 12 mm in the frequency, phase and slice direction respectively. The data was reconstructed to 2.0 mm × 2.0 mm × 6 mm, resulting in thirteen short axis slices.

### Calculation of parameters

T1 and T2 relaxation times are calculated from the five measurements by simulations of the longitudinal magnetizations Mz. In the absence of RF pulses, during the delay time between acquisitions, the M_z_ relaxes with a T1 relaxation time, approaching the unsaturated magnetization M_0_. In Figure 1, T1 relaxation occurs during the intervals M_3_-M_4_, M_5_–M_6_, M_7_–M_8_, M_9_–M_10_, M_11_–M_12_ and M_13_–M_1_. During this period each magnetization M_n+1_ can be calculated from a previous magnetization M_n_ with a time interval Δt according to:

1 $$ {M}_{n+1}={M}_0-\left({M}_0-{M}_n\right)\cdot {e}^{-\frac{\Delta t}{T_1}} $$

During acquisition, the observed T1 relaxation is altered in an effective T1* relaxation time by the RF pulses with a flip angle α and subsequent spoiling, repeated each repetition time T_r_. In Figure 1 T1* relaxation occurs during the intervals M_2_-M_3_, M_6_–M_7_, M_8_–M_9_, M_10_–M_11_ and M_12_–M_13_. The longitudinal magnetization approaches a saturated magnetization M_0_* which can be described by:

2 $$ \frac{{T_1}^{*}}{T_1}=\frac{{M_0}^{*}}{M_0}=\frac{1-{e}^{-\frac{T_R}{T_1}}}{1- \cos \left(\alpha \right)\cdot {e}^{-\frac{T_R}{T_1}}} $$

Again each magnetization M_n+1_ can be calculated from a previous magnetization M_n_ with a time interval Δt, but now according to:

3 $$ {M}_{n+1}={M_0}^{*}-\left({M_0}^{*}-{M}_n\right)\cdot {e}^{-\frac{\Delta t}{{T_1}^{*}}} $$

During the T1-sensitizing phase the longitudinal magnetization M_z_ is inverted. During the T2-sensitizing phase the longitudinal magnetization M_z_ is decreased by a factor ${e}^{-\frac{T{E}_{T2 prep}}{T_2}}$, where TE_T2prep_ is the time difference between the tip-down 90 degree RF pulse in x-direction and the tip-up 90 degree RF pulse in the –x-direction. The total acquisition results in 5 measured signals for each voxel, proportional to the magnetization M_2_, M_6_, M_8_, M_10_ and M_12_ in Figure 1.

The T1 and M_0_ values are found per voxel by using equation 1–3 in an iterative method by minimizing the squared difference between the expected magnetization M_z_ and the five signal data points. The T2 relaxation time is calculated using the extrapolated magnetizations just prior to the T2-sensitizing phase, M_1_, and just after the T2-sensitizing phase, M_2_, according to:

4 $$ {T}_2=-\frac{T{E}_{T2 prep}}{ \ln \left({M}_1/{M}_2\right)} $$

Generation of the T1 and T2 maps was implemented on a standalone version of SyMRI® (SyntheticMR, Sweden) and required less than 10 seconds on a standard PC.

### Validation

The MR pulse sequence was implemented on a Philips Ingenia 3T MR system (Philips Healthcare, Best, the Netherlands) and was evaluated by phantom studies as well as in-vivo comparison with other approaches.

#### Phantom studies

Test phantoms were manufactured with different concentrations of agarose (3-5% agarose in sterile water), doped with different amounts of gadolinium-Gadovist ([Gd] = 0.02-0.1 mM) each phantom thus representing specific relaxation times with T1 ~ 200–1800 ms and T2 ~ 35–170 ms. Phantoms were randomly distributed in a plastic container holding water at room temperature. A physiology simulator for artificial heart triggering was used and initially set to a heart rate of 60 beats per minute.

A series of 3D-QALAS scans were performed by intentionally varying some parameters that might affect the quantification, with the aim to investigate the robustness of the sequence. The simulated heart rate was varied from 40–120 bpm, the range previously recommended for cardiac relaxation times mapping [17], and the flip angle during acquisition, α, was varied from 4 – 8 degrees. Cardiac arrhythmias simulating atrial fibrillation or variations in the length of the cardiac cycle were deliberately applied to investigate its effect on the relaxation times measurements. Gaussian noise distributions with three different widths were used to change the length of the cardiac cycle in order to simulate fibrillation. Phantom measurements with simulated arrhythmia were repeated twenty times so that applied noise would occur randomly over the measurement.

Reference values of T1 were established by using a standard inversion recovery turbo spin-echo sequence, with inversion times (TI) varying from 100–5000 ms and a TR of 10000 ms. Reference values of T2 were established by using a standard multi echo turbo spin-echo sequence, with echo times (TE) varying from 10 ms to 200 ms by increments of 10 ms and a TR of 3000 ms.

### In vivo studies

Ten healthy volunteers with no history of cardiovascular disease underwent three 3D-QALAS scans, in the same session, in order to investigate the intra-scan variability of the method. The left ventricular images were viewed in short axis orientation. For comparison, three repeated conventional T1 and T2 measurements were performed in a mid ventricular 2D short axis slice. For T1 a 3-3-5 MOLLI [12] acquisition was performed and for T2 comparison a dual echo GraSE sequence was used, both with a resolution of 2.0 × 2.0 × 10.0 mm.

Ethics approval was granted and all subjects gave written informed consent. Details regarding the subjects are presented in Table 1.

**Table 1:** **Characteristics of subjects included in the study**

	**Sex**	**Age (years)**	**Height (cm)**	**Weight (kg)**	**Heart rate (bpm)**
Volunteer 1	Male	25	179	69	68
Volunteer 2	Male	31	193	85	49
Volunteer 3	Male	27	190	100	73
Volunteer 4	Male	48	182	77	50
Volunteer 5	Female	39	178	62	62
Volunteer 6	Male	46	183	85	61
Volunteer 7	Male	33	180	90	54
Volunteer 8	Male	30	188	82	59
Volunteer 9	Male	27	182	74	64
Volunteer 10	Male	29	181	65	86

Mean values and standard deviation of relaxation times for each individual were calculated from three repeated image acquisitions. In each examination, four regions of interests were defined (septal, anterior, lateral and posterior) in a mid cavity short axis slice. Mean values and standard deviations of relaxation times were also calculated for the separate myocardial regions, as an average of the ten subjects. For analysis of the T1 and T2 maps a standalone version of SyMRI® (Synthetic MR, Sweden) was used.

The 3D-QALAS sequence was also validated pre and post contrast in a 65 year old male with a myocardial infarction in the inferolateral region of the left ventricle. For comparison, late gadolinium enhancement imaging was acquired.

## Results

### Phantom studies

**Figure 2:**
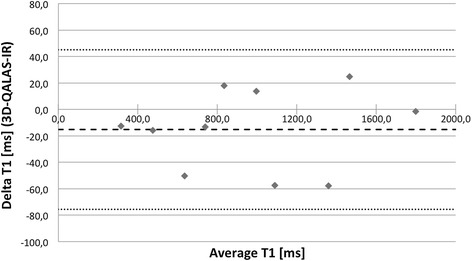
**Bland-Altman plot for T1 values at 60 bpm with 3D-QALAS and inversion recovery in phantoms.** Thick dashed line represents overall average difference between measurements (−15.3 ms), thin dashed lines represent 2SD.

**Figure 3:**
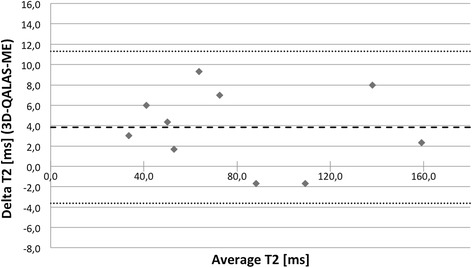
**Bland-Altman plot for T2 values at 60 bpm with 3D-QALAS and multi echo in phantoms.** Thick dashed line represents overall average difference between measurements (3.8 ms), thin dashed lines represent 2SD.

The relation between 3D-QALAS and Inversion Recovery for T1 respectively Multi Echo for T2 was investigated using Pearson correlation coefficient. There was a strong relation between 3D-QALAS and Inversion Recovery (R = 0.998; N = 10; p<0.01) and between 3D-QALAS and Multi Echo (R = 0.996; N = 10; p<0.01) for relaxation times in phantoms. Bland-Altman plots, in Figures 2 and 3, show the distribution of 3D-QALAS and Inversion Recovery respectively Multi Echo in phantoms. Average difference between the measurements was −15.3 ms for T1 and 3.8 ms for T2. The 2SD range was 60.3 ms for T1 and 7.5 ms for T2.

**Figure 4:**
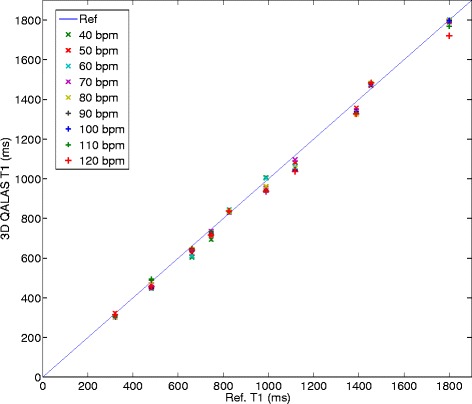
**Heart rate dependency in T1 measurement with 3D-QALAS.** Relationship between longitudinal relaxation time measurements with 3D-QALAS and the corresponding reference values measured with Inversion Recovery for different heart rates (40–120 bpm).

**Figure 5:**
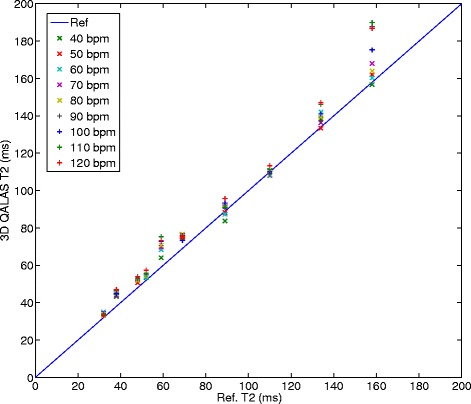
**Heart rate dependency in T2 measurements with 3D-QALAS.** Relationship between transverse relaxation time measurements with 3D-QALAS and the corresponding reference values measured with Multi Echo for different heart rates (40–120 bpm).

**Figure 6:**
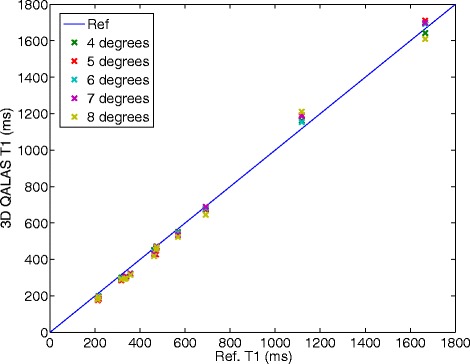
**Effect on T1 measurement using different radio frequency flip angles, α, during acquisition.** Measured longitudinal relaxation time (T1) with 3D-QALAS for different radio frequency flip angles, α, during data acquisition and corresponding reference values measured with Inversion Recovery. Radio frequency flip angles are varied from 4 degrees to 8 degrees.

**Figure 7:**
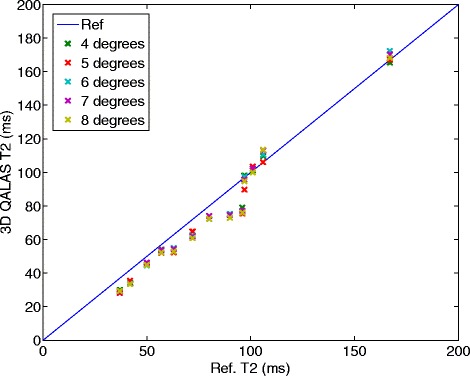
**Effect on T2 measurement using different radio frequency flip angles, α, during acquisition.** Measured transverse relaxation time (T2) with 3D-QALAS for different radio frequency flip angles, α, during data acquisition and corresponding reference values measured with Multi Echo. Radio frequency flip angles are varied from 4 degrees to 8 degrees.

The relationship between relaxation time measurement with 3D-QALAS and the corresponding reference values for different heart rates can be seen in Figure 4 for T1 and in Figure 5 for T2. The effect on T1 and T2 measurements when using different radio frequency flip angles during data acquisition, α, is shown in Figures 6 and 7.

**Figure 8:**
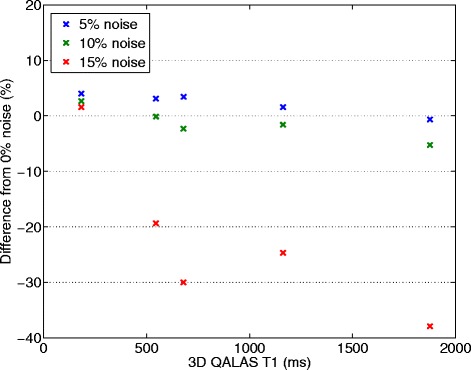
**Effect of deliberately applied arrhythmias on phantom T1 measurements.** Gaussian noise distributions with three different widths (5%, 10% and 15%) were used to change the length of the cardiac cycle, initially 60 beats per minute (bpm), in order to simulate atrial fibrillation. Deviation in percentage from measurements with 60 bpm and 0% noise can be seen as a function of T1 values measured with 3D-QALAS at a heart rate of 60 bpm with 0% noise.

**Figure 9:**
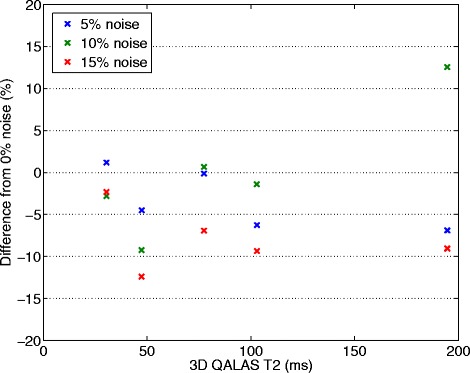
**Effect of deliberately applied arrhythmias on phantom T2 measurements.** Gaussian noise distributions with three different widths (5%, 10% and 15%) were used to change the length of the cardiac cycle, initially 60 beats per minute (bpm), in order to simulate atrial fibrillation. Deviation in percentage from measurements with 60 bpm and 0% noise can be seen as a function of T2 values measured with 3D-QALAS at a heart rate of 60 bpm with 0% noise.

Noise was deliberately applied to the cardiac cycle length of the phantoms to simulate arrhythmia. The results of twenty repeated measurements for each noise level (5% noise, 10% noise and 15% noise) are shown in Figure 8 for T1 measurements and in Figure 9 for T2 measurements. Mean deviation from a steady cardiac cycle length of 60 bpm was at a noise level of 5%: 2.3% for T1 and −3.32% for T2, at a noise level of 10%: −1.33% for T1 and −0.04% for T2 and at a noise level of 15%: −22.1% for T1 and −8.01% for T2.

### In vivo study

**Figure 10:**
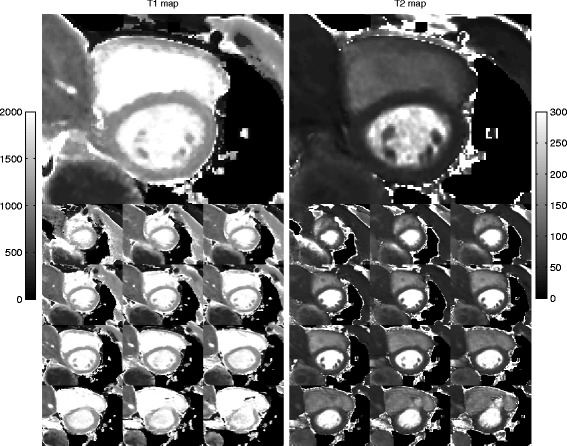
**3D-QALAS images from a healthy volunteer.** The thirteen 3D-QALAS short axis slices T1 maps (left) and T2 maps (right) of the left ventricular myocardium are shown. Slice 8 is shown on a larger scale. The gray scale indicates 0–2000 ms for T1 and 0–300 ms for T2.

Left ventricular myocardial relaxation times were successfully measured in-vivo using 3D-QALAS, MOLLI, and the dual echo GraSE sequence in all 10 healthy volunteers. The thirteen short axis slices of the left ventricular myocardium obtained with 3D-QALAS did not contain any noticeable artifacts or other imperfections, as demonstrated in Figure 10.

Left ventricular myocardial relaxation times, measured in-vivo with 3D-QALAS, were compared with their respective reference method and a good agreement was found, see Table 2. The mean value of T1 in healthy myocardium by 3D-QALAS was 1083 ± 43 ms (mean ± SD) and by MOLLI 1089 ± 54 ms. The mean value of T2 in healthy myocardium by 3D-QALAS was 50,4 ± 3,6 ms and by Dual Echo 50,3 ± 3,5 ms. Relaxation times were determined in healthy volunteers and derived from regions of interest such as the four regions of the short axis left ventricle that can be seen in Table 2. Values are based on three image acquisitions for each healthy volunteer. No significant difference in relaxation times was found between the different myocardial regions.

**Table 2:** **Myocardial relaxation times derived from regions of interest in healthy volunteers**

**Cases**	**Region**	**3D-QALAS T1**	**MOLLI**	**3D-QALAS T2**	**T2-DE**
		T1 (ms)	T1 (ms)	T2 (ms)	T2 (ms)
10 Healthy	Septal	1111 ± 46	1096 ± 62	51.1 ± 4.2	51.5 ± 4.4
	Anterior	1066 ± 35	1071 ± 53	49.3 ± 3.3	49.7 ± 3.0
	Lateral	1072 ± 46	1088 ± 43	50.2 ± 3.2	50.0 ± 3.3
	Posterior	1084 ± 32	1103 ± 54	50.9 ± 3.8	50.0 ± 3.0
	**Averages**	**1083 ± 43**	**1089 ± 54**	**50.4 ± 3.6**	**50.3 ± 3.5**

Mean values and standard deviations of myocardial relaxation times from ten healthy volunteers assessed in four regions within a short axis slice with 3D-QALAS, MOLLI and Dual Echo GraSE EPI.

**Figure 11:**
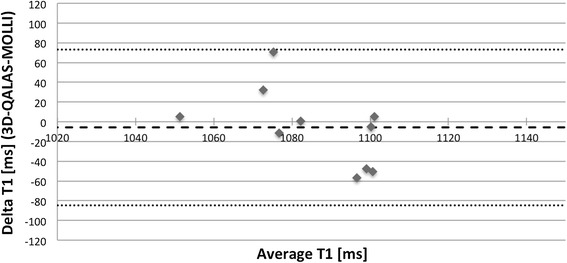
**Bland-Altman plot for native myocardial T1 values with 3D-QALAS and MOLLI.** Each healthy volunteer is represented with one measurement point. Thick dashed line represents overall average difference between measurements (−5,8 ms), thin dashed lines represent 2SD.

**Figure 12:**
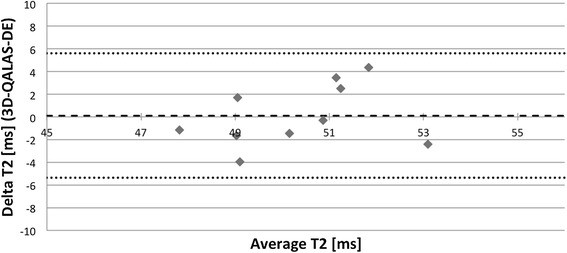
**Bland-Altman plot for native myocardial T2 values with 3D-QALAS and Dual Echo.** Each healthy volunteer is represented with one measurement point. Thick dashed line represents overall average difference between measurements (0,1 ms), thin dashed lines represent 2SD.

Bland-Altman plots, Figures 11 and 12, show the distribution of 3D-QALAS and corresponding reference methods in ten healthy volunteer. Average difference between the measurements was −5.8 ms for T1 and 0.1 ms for T2. The 2SD range was 78.9 ms for T1 and 5.5 ms for T2.

**Figure 13:**
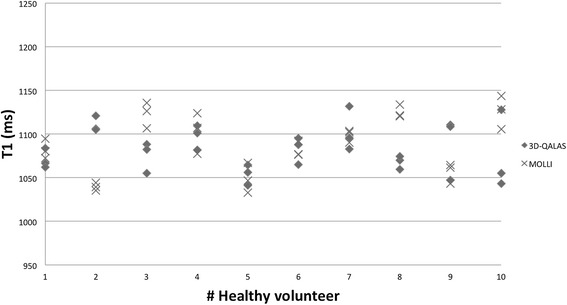
**Individual myocardial T1 values from 3D-QALAS and MOLLI.** Values are derived from four regions of interests (septal,anterior,lateral and posterior) in a mid-cavity short axis slice from three repeated measurements and are displayed as mean values representing each measurement.

**Figure 14:**
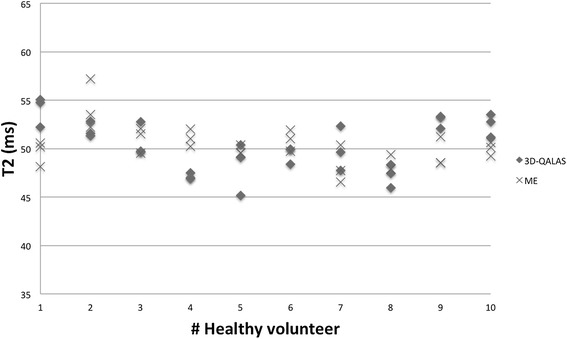
**Individual myocardial T2 values from 3D-QALAS and Two-Point Multi Echo (ME).** Values are derived from four regions of interests (septal, anterior, lateral and posterior) in a mid-cavity short axis slice from three repeated measurements and are displayed as mean values representing each measurement.

**Figure 15:**
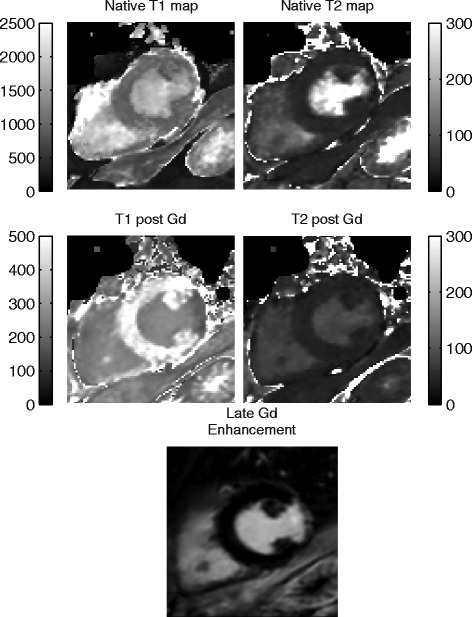
**Native and post contrast images from a 65 years old male with a lateral myocardial infarction.** Pre and post contrast 3D-QALAS maps together with a conventional late gadolinium enhancement image illustrates the area of infarction.

Results of individual myocardial relaxation times for the ten healthy volunteers investigated with 3D-QALAS and the corresponding in-vivo reference methods are shown in Figure 13 for T1 and in Figure 14 for T2. A two-tailed unpaired Student T-test was used to calculate whether the differences between population means were significant. No significant difference in relaxation times was found between 3D-QALAS and MOLLI (N = 10; p = 0.65) respectively 3D-QALAS and Dual Echo (N = 10; p = 0.925). Pre and post contrast 3D-QALAS maps together with a conventional late gadolinium enhancement image from a patient with a myocardial infarction can be seen in Figure 15.

## Discussion

In order to make myocardial relaxation time mapping more clinically applicable in a daily routine, improvements in speed and coverage with maintained accuracy and precision are desirable. In this work we have developed a fast and accurate method providing simultaneous acquisition of T1 maps and T2 maps with coverage of the entire left ventricular myocardium in a single breath hold.

Relaxation time measurements with 3D-QALAS in phantoms showed highly accurate results over a large range of T1 and T2 values, covering both expected contrast enhanced and native conditions of myocardial tissue. The method appeared to be robust for changes in flip angle, heart rate, and arrhythmia during the measurement.

Performing relaxation time mapping in a whole 3D volume instead of in a 2D slice is followed by the requirement to collect larger quantity of k-space data in one breath hold. To fulfill this criterion without increasing the total acquisition time, i.e. the length of the breath hold, implies that there can only be a limited number of data points acquired. For the T1 curve only 4 points were measured, resulting in a dynamic range for 3D-QALAS that lies at least in the range 200–1800 ms for T1 as shown in the phantom study. Especially for the low T1 values it was important to use the simulated magnetization evolution for the analysis, rather than an exponential fit. In this analysis a perfect inversion pulse is assumed, which essentially adds a data point to the measurement: the magnetization right after the inversion pulse must be equal to minus the extrapolated magnetization just prior to the inversion pulse. This assumption makes the analysis more stable compared to the exponential fit as for example is used in the MOLLI analysis. Furthermore the 3D-QALAS method needs an acquisition read out time that is slightly longer, < 230 ms, together with a larger slice thickness, 12 mm, than existing 2D relaxation time mapping methods [12,13,18] to be able to sample the larger quantity of data required to cover the whole 3D volume and to maintain good in-plane spatial resolution with a high image quality.

The dynamic range of T1 in 3D-QALAS can also be related to that of saturation recovery and inversion recovery based T1-mapped methods. In 3D-QALAS the magnetization just prior to the inversion pulse is not fully relaxed since it is placed at only one cardiac cycle after the T2 preparation pulse. The magnetization is therefore more likely to be in the interval 0.5 – 1.0 times the unsaturated magnetization and hence in the interval −0.5 - −1.0 after the inversion. This places the dynamic range of T1 of 3D-QALAS somewhere between saturation recovery and inversion recovery based T1-mapping methods.

The results also showed that the T2 measurements of 3D-QALAS were valid in the range of at least 35 – 170 ms. The expected dynamic range for T2 can be estimated by setting requirements on the signal intensity of the exponential decay curve during the T2 sensitizing phase. The upper limit of T2 can be defined by requiring that the signal intensity should at least have decreased to 90% at the end of the measurement (corresponding to ${e}^{-\frac{TE}{T2}}=0.9$). The lower limit of T2 can be defined by requiring that the signal intensity should at most have decreased to 10% (corresponding to ${e}^{-\frac{TE}{T2}}=0.1$) at the end of the measurement, such that the signal does not disappear in the noise level. Using these requirements, in combination with the chosen TE = 50 ms, the expected dynamic range of T2 values in our experiment becomes [22–475] ms, which is larger than the evaluated range.

The gradient echo sequence used for quantification could either be low flip-angle spoiled gradient echo, as suggested for 3D-QALAS, or high flip-angle balanced SSFP, as common in the MOLLI approach. As off-resonance effects may be challenging in 3D application with bSSFP, a low-flip angle approach is chosen in 3D-QALAS. The penalty of using spoiled gradient echo is the intrinsically lower signal strength compared to bSSFP. The penalty in SNR by choosing a low flip-angle spoiled gradient echo should however be weighted against the benefits of using a true 3D acquisition, including a full coverage of the heart, and less tissue moving in and out of the excited volume.

Quantification methods can be sensitive to inhomogeneity in the radio frequency field strength (B1), which would affect the actual local flip angle. This makes the flip angle dependency of quantification techniques relevant. The results shows that the 3D-QALAS sequence was insensitive to RF flip angles changes in the range of 4° - 8°, which can be interpreted as the relaxation time calculation is relatively insensitive to changes in the B1 field.

Heart rate dependency has proven to be a challenge for myocardial T1 mapping methods, especially at long longitudinal relaxation times [19]. We did not see such dependence within the tested range of heart rate with the method proposed in this work, either for T1 measurements or for T2 measurements, as can be seen in Figures 4 and 5. This may be a result of the use of a simulation of the full magnetization evolution during the acquisition, which included saturation effects due to long T1 relaxation times in comparison to one heartbeat. This seems to be a more robust approach than using the assumption that the magnetization has returned to M0 after a certain delay time. In theory, the dynamic range might be affected by heart hate. The dynamic range becomes eventually slightly lower at higher heart rates than for lower heart rates since the recovery of the magnetization vector is less when the total acquisition time is shorter. This assumed loss of dynamic range was not evident in the volunteer with the highest heart rate, 86 beats/min. The proposed method is robust to small random changes in cardiac cycle lengths, as was done in phantoms to simulate cardiac arrhythmia. Gaussian noise distributions applied to the length of the cardiac cycle of 5% and 10% has a minor effect on the quantification of relaxation times. The error seen at an applied noise level of 15% deviation from a steady cardiac cycle length is most probably related to the exclusion of extremely short cardiac cycles. Cardiac cycles longer than the set value on the scanner usually pose no problem for the data acquisition to be completed within the expected cardiac cycle length, other than motion artifacts if the data acquisition does not occur at the right moment in the cardiac cycle. However, cardiac cycles much shorter than the set value will result in incomplete data and the need to complete the acquisition in the next cardiac cycle. This will thus introduce a fault in the calculation of relaxation times. To overcome this problem, a higher heart rate than the actual mean heart rate can be set on the scanner.

Left ventricular myocardial relaxation times with 3D-QALAS were compared with a 3-3-5 MOLLI for T1 measurement, a dual echo GraSE sequence for T2 measurement and consistent results were found between 3D-QALAS and the corresponding reference method. In-vivo relaxation time measurements with 3D-QALAS were in accordance with previously published results for both T1 and T2 [13,20]. Reference values for healthy myocardial tissue seem to differ between manufacturers and between different quantification methods. According to a recently published study [21], several T1-mapping methods suffer from poor absolute accuracy and reference values from different relaxation time mapping methods might thus be difficult to compare with each other. Native T1 values at 3T from inversion recovery based T1 mapping methods have shown a spread from < 900 ms to > 1300 ms in healthy volunteers [22,23].

We have evaluated the accuracy and intra-scan variability of 3D-QALAS using phantom studies and in-vivo studies on healthy volunteers. The results are promising, but further evaluation post-contrast and in cardiac disease is necessary before assessment of extracellular volume or application of the method in larger clinical studies. One patient with myocardial infarction is however included in the study to demonstrate the method at short T1, post-contrast.

The presented original version of 3D-QALAS provides 13 slices of the left ventricular volume within a breath hold of 15 heartbeats. The sequence can be modified to a shortened version, in which k-space is covered in two segments instead of three segments as in the original version. We have successfully tested a version covering eight slices within a breath hold of 10 heartbeats, still giving the same resolution as the original version but thus with less ventricular coverage. This modified version enables use of this method in patients having difficulty holding their breath and gives the possibility of full coverage of left ventricular volume in two breath holds.

3D-QALAS enables relaxation time mapping of both T1 and T2 in a 3D volume in a single breath hold. Moreover, the T1 and T2 maps are inherently co-registered, avoiding post-processing registration errors. It is reasonable to believe that quantitative information from both these parameters facilitates better differentiation between myocardium affected by e.g. edema, fibrosis, storage diseases and normal myocardium.

## Conclusions

The 3D-QALAS principle has demonstrated good accuracy and intra-scan-variability both in-vitro and in-vivo. It allows a rapid acquisition and provides quantitative information of both T1 and T2 relaxation times in the same scan with full coverage of the left ventricle, enabling the method to be clinically applicable to a broader spectrum of cardiac disorders.
